# A Comprehensive Review of the Phytochemical Constituents and Bioactivities of *Ocimum tenuiflorum*

**DOI:** 10.1155/2024/8895039

**Published:** 2024-10-22

**Authors:** Keshab Bhattarai, Rabin Bhattarai, Ram Darash Pandey, Babita Paudel, Hari Datta Bhattarai

**Affiliations:** ^1^Central Department of Chemistry, Tribhuvan University, Kirtipur, Kathmandu, Nepal; ^2^Natural Product Chemistry, Center for Natural and Applied Sciences, Kathmandu, Nepal; ^3^Department of Chemistry, Amrit Campus, Thamel, Kathmandu, Nepal; ^4^Central Department of Botany, Tribhuvan University, Kirtipur, Kathmandu, Nepal

**Keywords:** anticancer, antidiabetic, luteolin, *Ocimum tenuiflorum*, oleanolic acid, rosmarinic acid

## Abstract

*Ocimum tenuiflorum*, commonly known as Tulsi, is revered in Ayurveda for its extensive medicinal properties. However, there is a need to consolidate current knowledge on its phytochemical constituents and their pharmacological activities to identify potential areas for further research and drug development. This review aims to bridge this gap by providing a comprehensive analysis of the bioactive secondary metabolites found in *O. tenuiflorum*, such as rosmarinic acid, oleanolic acid, luteolin, ursolic acid, and limonene, and their associated therapeutic effects. The review will highlight the pharmacological importance of these metabolites, which exhibit antioxidant, neuroprotective, anticancer, and anti-inflammatory properties. Additionally, this study will explore the plant's wide range of beneficial qualities, including anti-inflammatory, antioxidant, anticholinergic, pain-relieving, antimicrobial, stress-reducing, antidiabetic, anticancer, liver-protective, ulcer-inhibiting, antifungal, and wound-healing attributes. Furthermore, this review focuses on the plant's potential in treating conditions such as asthma, persistent fever, tuberculosis, malaria, skin discoloration, itching, digestive issues, hemorrhoids, bone fractures, gout, urinary tract infection, and diabetes. By reviewing the current literature, the aim is to identify the gaps in the existing research and propose directions for future studies. This comprehensive review will serve as a valuable resource for researchers in the development and investigation of novel drugs derived from *O. tenuiflorum*.

## 1. Introduction

From ancient times, different parts of plants (roots, stems, leaves, flowers, seeds, and barks) have been used as medicine for the treatment of several ailments [1]. Since then, plants have served as a remarkable source of compounds of medicinal value. Many people in developing countries rely on medicinal plants because they are abundant and affordable and have no side effects [2]. One such sacred plant of high ethnomedicinal value is *Ocimum tenuiflorum* [3]. This aromatic medicinal herb belongs to the family Lamiaceae. In 1753, Linnaeus described the genus *Ocimum* including five species. However, these days, more than 50 species have been identified with potential therapeutic importance [3, 4]. The species of *Ocimum* are distributed all over the tropical and subtropical regions. Due to their therapeutic and economic importance, the cultivation of some species is undertaken [5]. This plant exhibits many medicinal properties such as antioxidant, antidiabetic, anti-inflammatory, anticancer, antinociceptive, antifertility, anthelmintic, cardioprotective, and antimicrobial [6]. Most species of Ocimum are used to treat disease and functional disorders such as diabetes, dysentery, hemorrhoids, diarrhea, constipation, coughs, tuberculosis, eye and ear complaints, stomach disorder, abdominal pains, headaches, febrile illness, malaise, soreness, fever, reducing swelling, and central nervous system disorder [4, 7, 8]. Among them, *O. tenuiflorum* is one of the most important species. *Ocimum tenuiflorum* is also known as Tulasi or Tulsi in Nepali and Hindi and Holy Basil in English. *Ocimum tenuiflorum* is mainly native to tropical and subtropical regions [9]. The name “Tulsi” is derived from Sanskrit, and it means “incomparable one” [10]. In Ayurveda, the aromatic medicinal plant Tulsi is often referred to as the “elixir of life” [11]. It is a branched perennial herb, which can grow up to 1 m tall, and possesses an aromatic odor, with some woody tissue at the stem bases. The leaves are broadly elliptical and pubescent on their surfaces, measuring 3–6 cm in length and 1–2.5 cm in width. The flowers are terminal and form slender racemes or panicles. The stems are four-angled, purplish, and hairy [12–14]. Because of its therapeutic value, the entire plant can be utilized for medical treatment, and it is often referred to as the “Queen of Plants” [10, 15]. This plant produces a variety of volatile oils, including terpenes, phenol, and aldehydes. Additionally, the plants are said to contain tannins, alkaloids, saponins, and glycosides [16]. Due to the presence of natural products, the plant possesses diverse biological activities such as cardioprotective, antidiabetic, antimicrobial, hepatoprotective, antifertility, antifungal, anticancer, stomachache, headaches, common colds, inflammation, analgesics, antiemetics, antipyretics, and stress reducers [17–19]. The primary objective of this review is to explore the chemistry of *O. tenuiflorum*, including the bioactivity of its metabolites and extracts in various solvent mediums. Traditionally, this plant has been employed for numerous therapeutic purposes in different countries as shown in Table 1, yet its chemical composition remains largely undocumented. The presence of secondary metabolites spanning different classes constitutes a pivotal factor contributing to the plant's substantial therapeutic significance. There exists an extensive body of the literature on the phytochemical and ethnomedical uses of *O. tenuiflorum*. Consequently, this article provides an in-depth review of the phytochemical constituents of *O. tenuiflorum* and their pharmacological activities. By systematically analyzing these metabolites and their therapeutic potential, the study highlights the plant's significance in modern pharmacology and its potential as a source of novel drug candidates.

## 2. Methodology

To gather information on the ethnomedicinal uses, in vivo and in vitro biological activities, metabolites found in *O. tenuiflorum*, and the biological activities of its metabolites, we conducted a comprehensive search across various databases, including Google Scholar, Research gate, Web of Science, PubMed, SciFinder, Wiley Online Library, Science Direct, Springer, Taylor and Francis, Elsevier, Chemical Abstracts, and Scopus. Keywords such as antioxidants, antimicrobial, anti-inflammatory, anticancer, antistress, secondary metabolites, natural products, and phytochemicals of *O. tenuiflorum* (also known as *O. sanctum*) were used to explore the chemistry of the plant. We selected standard articles that provided sufficient pharmacological and ethnomedicinal insights. A total of 226 articles from 1987 to 2024 were reviewed to extract relevant information for the comprehensive evaluation of *O. tenuiflorum* pharmacological and phytochemical properties. These articles were chosen based on the biological activities of *O. tenuiflorum* and its available metabolites. The selection process also took into consideration a comparative study with similar species, as well as the identification of compounds using advanced analytical tools and advanced assays used to evaluate the biological activities.

## 3. Phytochemical Constituents

### 3.1. Flavonoids

**Figure 1:**
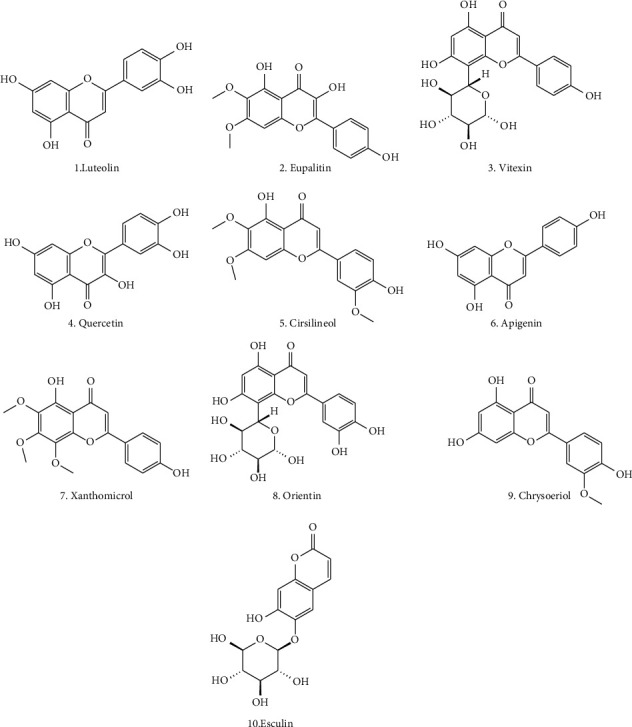
Some bioactive flavonoids from the *Ocimum tenuiflorum*.

Flavonoids are the most abundant phytochemicals with low-molecular-weight polyphenol structures [30, 31]. *Ocimum tenuiflorum* contains different kinds of flavonoids, which are responsible for the plant's therapeutic activity as mentioned in Table 2. Some flavonoids that are commonly present in *O. tenuiflorum* are luteolin, apigenin, eupalitin, xanthomicrol, genkwanin, demethylnobiletin, salvigenin, luteolin-7-O-glucuronide, isoorientin, orientin, galuteolin, apigenin-7-O-glucuronide, kaempferol, kaempferide, chrysoeriol, isosakuranetin, vitexin, isovitexin, quercetin, cirsimaritin, chrysoeriol, cirsilineol, isothymusin, molludistin, vicenin, luteolin-5-glucoside, esculin, robinetintrimethyl ether, and esculetin [32–36]. The structures of key bioactive flavonoids, which are particularly prominent in *Ocimum* species, are shown in Figure 1.

### 3.2. Phenols and Phenolic Acids

**Figure 2:**
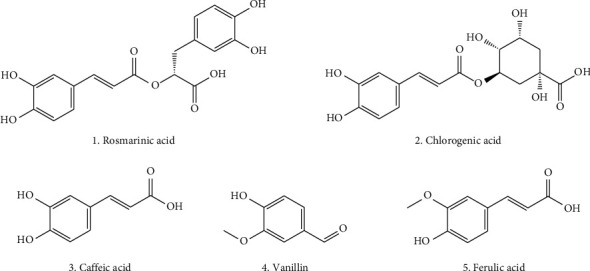
Some bioactive phenolics from the *Ocimum tenuiflorum*.

Phenolic metabolites include phenols and phenolic acid. Phenols and phenolic acid are therapeutically important metabolites as they act as antioxidants and are responsible antisickling, antiosteoporotic, anticarcinogenic, and other bioactivity as mentioned in Table 3 [75, 76]. The shikimic acid and phenylpropanoid pathways serve as the synthetic routes for phenolic compounds [77]. *Ocimum tenuiflorum* is a well-known plant for its antioxidant properties due to the presence of several phenolic compounds. Few of them are rosmarinic acid, (E)-*p*-coumaroyl 4-O-*β*-D-glucoside, chlorogenic acid, caffeic acid, vanillin, methylisoeugenol, vanillic acid, sinapic acid, *p*-coumaric acid, 3-(3,4-dihydroxyphenyl) lactic acid, protocatechuic acid, 3,4-dimethoxycinnamic acid, *p*-hydroxybenzoic acid, ferulic acid, and bieugenol [33, 34, 78]. The structures of key bioactive phenolic metabolites, which are particularly prominent in *Ocimum* species, are shown in Figure 2.

### 3.3. Triterpenoids and Steroids

**Figure 3:**
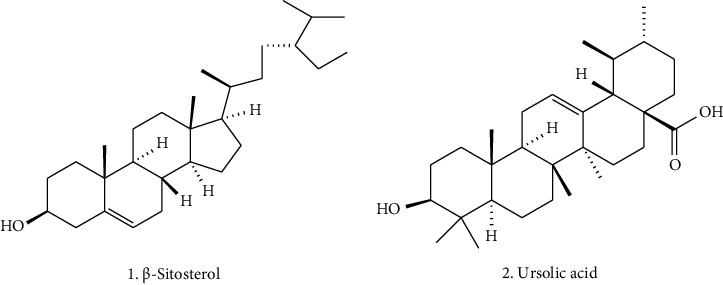
Some bioactive triterpenoids from the *Ocimum tenuiflorum*.

Triterpenoids are important secondary metabolites present in animals and plants that possess immense pharmaceutical importance [108]. In living bodies, triterpenoids are considered the building block from which steroids are formed [109]. Steroids are well known for their biological activities related to growth-regulating activity in plants, and anti-inflammatory and immune-modulating properties [110, 111]. Small changes in the steroids may lead to significant biological changes. *Ocimum tenuiflorum* consists of multiple triterpenoids and steroids, which bear important therapeutic importance as shown in Table 4. Few of them are *β*-sitosterol, ursolic acid, trihydroxyursolic acid, *β*-sitosterol-3-O*β*-D-glucopyranoside, oleanolic acid (OA), stigmasterol, campesterol, ocimic acid, urs-12-en-3*β*,6*β*,20*β*-triol-28-oic acid, and 16-hydroxy-4,4,10,13-tetramethyl-17-(4-methyl-pentyl)-hexadecahydrocyclopenta [*α*] phenanthrene-3-one [36, 112–117]. The structures of key bioactive triterpenoids, which are particularly prominent in *Ocimum* species, are shown in Figure 3.

### 3.4. Monoterpenes

**Figure 4:**
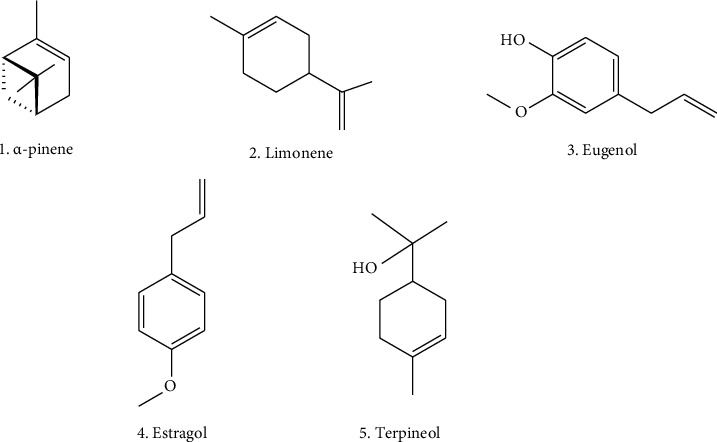
Some bioactive monoterpenes from the *Ocimum tenuiflorum*.

Monoterpenes are organic compounds present in the essential oils of plants, contributing to the plants' flavor and aroma, and play a significant role in various biological applications as shown in Table 5, particularly in the development and design of drugs [126, 127]. Some important monoterpenes reported in *O. tenuiflorum* are *α*-pinene, camphene, sabinene, *β*-pinene, 1,8-cineole, *β*-trans-ocimene, camphor, borneol, tricyclene, myrcene, phellandrene, terpinene, limonene, ocimene, terpinolene, sabinene hydrate, carene, fenchone, linalool, camphene hydrate, terpinen-4-ol, terpineol, estragole, and eugenol [18, 128–130]. The structures of key bioactive monoterpenes, which are particularly prominent in *Ocimum* species, are shown in Figure 4.

### 3.5. Sesquiterpenes

Sesquiterpenes are promising secondary metabolites with pharmaceutical importance. *Ocimum tenuiflorum* consists of a number of sesquiterpenes. Few of them are copaene, zingiberene, bourbonene, guaiene, bergamotene, sesquiphellandrene, farnesene, sesquisabinene, humulene, bicyclogermacrene, germacrene, bisabolene-(Z), *δ*-cadinene, *α*-bisabolene, amorphene, caryophyllene oxide, *γ*-muurolene, *α*-muurolene, *α*-cadinol, bourbonene, *γ*-cadinene, *α*-caryophyllene, *β*-caryophyllene, germacrene D, *β*-guaiene, *α*-longipinene, *α*-panasinsen, selina-6-en-4-ol, nerolidol, spathulenol, aromadendrene oxide, *α*-calacorene, 1-4-cadinadiene, *β*-bisabolene, alloaromadendrene, *β*-gurjunene, *β*-cubebene, *β*-elemene, and *γ*-eleneme [18, 128–130]. These metabolites are mostly found in the essential oils of the plant and possess multiple biological activities. More specifically, sesquiterpenes have shown pharmacological activities such as, antimicrobial, antifeedant, immunomodulatory, anti-inflammatory, antitumor, and antimalarial [150].

### 3.6. Esters, Aldehyde, and Ketone

Ester, aldehyde, and ketones are organic compounds containing different functional groups. *Ocimum tenuiflorum* consists of several esters, aldehydes, and ketones, which possess significant biological activities. Some of them are methyl isovalerate, ethyl isovalerate, pentanal, hexane-3-one, 4-methyl-4-hepten-3-one, and octyl ester [18, 114, 151, 152]. Some compounds belonging to this group possess pharmacological importance such as antioxidant, antibacterial, antifungal, and anticancer, but good literature is lacking on the reported compounds.

### 3.7. Other Secondary Metabolites

**Figure 5:**
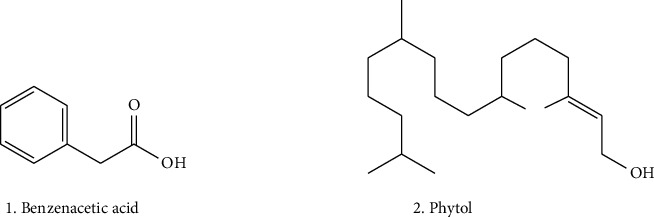
Some bioactive metabolites from the *Ocimum tenuiflorum*.

There are different classes of metabolites present in various parts of the *O. tenuiflorum*, ranging from aliphatic alcohol to complex compounds such as lumiflavine. These are included in another category of metabolites, and these metabolites exhibited crucial biological application as shown in Table 6. A few examples are sotolon, hexane-2-ol, benzene-1,2-dicarboxylic acids, benzeneacetic acid, lumiflavine (reported as lumiflavine), phytol, and 1,4-cyclohexadiene [114, 151, 152]. The structures of metabolites, which are crucial for biological applications, are presented in Figure 5.

## 4. Biological Activities of *Ocimum tenuiflorum*

### 4.1. Antioxidant Activity

*Ocimum tenuiflorum* is a well-known potential source of antioxidants. Saravanan et al. evaluated the antioxidant property of *O. tenuiflorum*, i.e., Tulsi. The DPPH scavenging results indicated that at higher concentrations of 200–500 *μ*g/mL, Tulsi exhibited strong antioxidant properties [160]. Similarly, Chaudhary et al. calculated the antioxidant properties of the sample in different solvent mediums by various methods [33]. In this study, n-butanol fraction was most effective in inhibiting DPPH radical, ABST radical, and phosphomolybdate, with EC_50_ values 3.91 ± 0.3 µg/mL, 1.6 ± 0.1 µg/mL, and 2.31 ± 0.1 µg/mL, respectively, whereas methanolic extract was most effective to inhibit the hydroxyl radical with an EC_50_ value of 5.30 ± 0.43 *μ*g/mL [33]. Among the different species of the same genus, Agarwal reported that the ethyl acetate fraction of *O. tenuiflorum* has demonstrated a strong antioxidant capacity compared to the same solvent fraction of *O. kilimandscharium* [112]. Rindhe performed the antioxidant activity of Tulsi using two different methods, and among them Tulsi exhibited strong inhibition for DPPH and hydrogen peroxide. For DPPH at 100 *μ*g/mL, Tulsi exhibited 80.19% inhibition, and at the same concentration for hydrogen peroxide, it exhibited 39.92% inhibition, which is stronger compared to ascorbic acid [161]. In addition, the in vivo analysis of *O. tenuiflorum* demonstrated it as a potential source of an antioxidant. Ramesh and Satakopan conducted the antioxidant activity of *O. tenuiflorum* against toxicity induced by Cadmium in rats [162]. Lipid peroxidation levels were shown to have significantly decreased following the oral treatment of *O. tenuiflorum* at doses of 100 and 200 mg/kg body weight, both before and after cadmium-induced toxicity, respectively. Lipid peroxidation levels had previously increased following the oral administration of 6.0 mg/kg body weight CdCl_2_. It also significantly increased the levels of catalase, reduced glutathione, glutathione peroxidase, superoxide dismutase, and vitamin C [161]. The mechanism behind the escalation of reduced glutathione is by diminishing the oxidative free radical by donating H. This increase in reduced glutathione helps to enhance the level of glutathione peroxidase in the liver [162]. Moreover, the oral administration of *O. tenuiflorum* before and after 10 mg/kg body weight increases the level of reduced glutathione, lowers the level of lipid peroxidation, and helps to alter the activities of serum glutamate, oxaloacetate, transaminase, and serum glutamate, pyruvate, and transaminase [162]. This was reported in Sharma et al.'s study on the toxicity induced by mercury in Swiss albino mice, which increased the levels of lipid peroxidation, serum glutamate, oxaloacetate, transaminase, serum glutamate, and pyruvate transaminase. The in vivo and in vitro antioxidant potential of *O. tenuiflorum* was ascribed to the presence of several bioactive phytochemicals.

**Figure 6:**
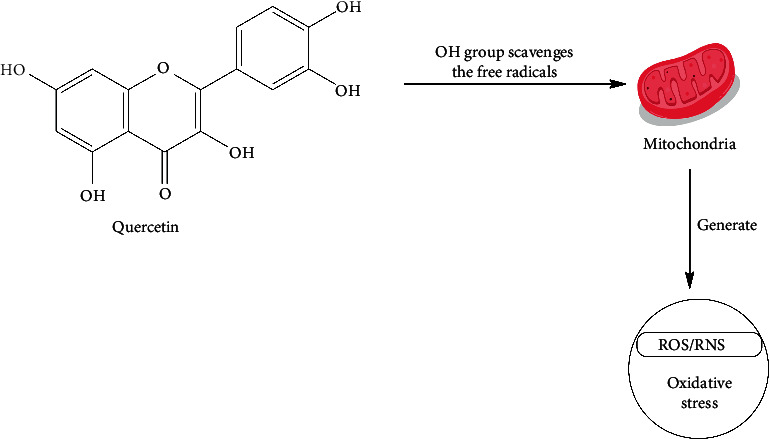
Function of quercetin as an antioxidant [163].

The antioxidant property of *O. tenuiflorum* is due to the presence of metabolites such as caffeic acid, quercetin, luteolin, and eugenol. For reference, quercetin due to the presence of the hydroxyl group and its unique position can interact with various signal transduction pathways by either activating, inhibiting, upregulating, or downregulating numerous body molecules. This action helps enhance the body's antioxidant capacity and repair damage. Along with the mitochondrial electron transport chain, environmental factors can increase the production of reactive oxygen species (ROS). Quercetin regulates both enzyme-mediated and nonenzyme-dependent antioxidant defense systems, and the general process is shown in Figure 6. It also modulates the signal pathways such as NRFB, AMPK, and MAPK, which are influenced by ROS, to bolster the antioxidant defense system and maintain oxidative balance.

### 4.2. Antimicrobial Activity

*Ocimum tenuiflorum* has been extensively studied for its antimicrobial properties. The plant has demonstrated a strong inhibition against Gram-positive and Gram-negative bacteria. Dixit et al. studied the antibacterial activity of *O. tenuiflorum* at different concentrations of ethanolic, methanolic, and aqueous extracts against *Bacillus subtilis*. The results indicated that the methanolic extract was more effective compared to other solvent extracts as it showed inhibition of 2 and 5 mm at even 0.2 and 0.3 g/mL concentrations, respectively [164]. Similarly, Mahmood et al. reported the essential oil obtained from *O. tenuiflorum* showed strong inhibition against various Gram-negative and Gram-positive bacteria, including *Escherichia coli*, *P. aeruginosa*, *Klebsiella* sp., *Proteus mirabilis*, and *S. aureus*, with zones of inhibition measuring 15.4, 17.8, 20, 20, and 41.5 mm, respectively [165]. *Ocimum tenuiflorum* comprises antifungal agents to inhibit fungal pathogens. Sivareddy et al. conducted the antifungal activity of *O. tenuiflorum* leaf against *Candida albicans*. Both the ethyl acetate and ethanolic extract of the plant exhibited the same zone of inhibition and minimum inhibitory concentration (MIC) against the tested organism, i.e., 13 mm and 2000 *μ*g/mL, respectively [166]. Piras et al. also conducted the antifungal activity of essential oil of two species of Ocimum, *O. basilicum* and *O. tenuiflorum*. *Ocimum tenuiflorum* essential oil was found to be the most effective against the tested species of *C. albicans*, *C. tropicalis*, *C. krusei*, *C. guilliermondii*, *C. parapsilosis*, *Cryptococcus neoformans*, *T. mentagrophytes*, *Trichophyton rubrum*, *T. verrucosum*, *Microsporum canis*, *M. gypseum*, and *Epidermophyton floccosum*. For *Candida* spp., *Cr. neoformans*, and *dermatophytes*, the MICs and minimum lethal concentrations (MLCs) were, respectively, 0.16 and 0.64 *μ*g/mL, 0.32, and 0.32–0.64 *μ*g/mL, 1.25–2.5 and 0.64 *μ*g/mL. Eugenol and methyl eugenol, two metabolites of *O. tenuiflorum* that showed high antifungal activity against the aforementioned species, are responsible for the plant's potent antifungal action [129].

**Figure 7:**
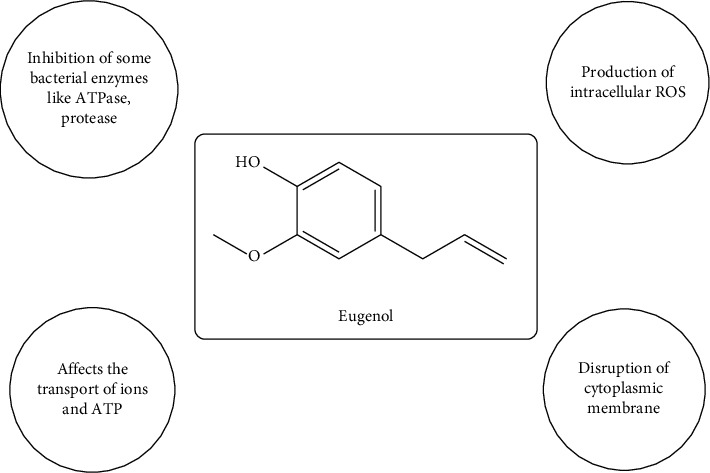
Mechanism of eugenol as an antibacterial agent.

Another study was conducted by Balakumar et al. against clinically isolated dermatophyte fungi and observed that the alcoholic and aqueous extract and fractions illustrated strong antifungal activity [167]. The bioactive compounds isolated from *O. tenuiflorum*, particularly flavonoids, alkaloids, and essential oils, have antimicrobial properties. Metabolites such as eugenol, estragole, ursolic acid, and ferulic acid are well documented for their antimicrobial activity. Eugenol exerts its antibacterial activity through several mechanisms as shown in Figure 7. It penetrates bacterial cell membranes, particularly in Gram-negative bacteria, causing structural alterations that lead to the leakage of intracellular components and ultimately cell death. Additionally, eugenol inhibits crucial bacterial enzymes, such as proteases and membrane-bound ATPases, disrupting essential metabolic processes. Furthermore, it induces oxidative stress by generating ROS, which damage cellular components, including DNA, proteins, and lipids [168, 169].

### 4.3. Antidiabetic Activity

Secondary metabolites found in *O. tenuiflorum* were reported to inhibit the *α*-glucosidase enzyme, which is a key enzyme responsible for catalyzing carbohydrate digestion. For the treatment of type 2 diabetes, *α*-glucosidase inhibitors are used. These drugs impede the absorption of carbohydrates [170]. Leaves of *O. tenuiflorum* are well known to lower blood glucose levels. Sethi et al. studied the antidiabetic activity of leaves of *O. tenuiflorum* and reported that chewing the leaves 2-g/kg body weight for the subsequent 30 days led to lower blood glucose levels in the tested group [171]. Rao et al. also conducted a comparative study of ethanolic extract of *O. tenuiflorum* with glibenclamide; with regular administration of the ethanolic extract, the level of blood glucose reduced abruptly in the hyperglycemic rats. When the ethanolic extract was administered, the results were comparable to those obtained with standard drugs. There was a 51.5% reduction in blood glucose levels on the third day and a 52% reduction in fasting blood glucose levels on the tenth day with the use of standard drugs. Similarly, there was a 50% reduction in blood glucose levels on the third day, and a 45% reduction in fasting blood glucose levels on the tenth day with the use of the ethanolic extract on the alloxan induced diabetes rats [172]. Mousavi, Salleh, and Murugaiyah discovered the interesting results on the in vitro analysis of *α*-amylase and *α*-glucosidase inhibition activity. The results indicated that ethyl acetate–butanol and ethanol–water fractions of *O. tenuiflorum* leaves exhibited less IC_50_ values of 0.59 ± 0.03 mg/mL and 1.45 ± 0.04 mg/mL, respectively, for *α*-amylase. The values were 0.05 ± 0.00 mg/mL and 0.10 ± 0.00 mg/mL, respectively, for *α*-glucosidase. The values for standard acarbose are IC_50_ 1.54 ± 0.21 mg/mL and 0.36 ± 0.21 mg/mL for *α*-amylase and *α-glucosidase,* respectively [173]. Parasuraman et al. reported the hydroalcoholic extract of *O. tenuiflorum* demonstrated significant antidiabetic and anti-hyperlipidemic effects in diabetic rats induced by STZ and NIC when administered at doses of 250 and 500 mg/kg body weight. It reduced the glucose levels from 229.80 ± 10.00 to 129.00 ± 13.20 [174].

**Figure 8:**
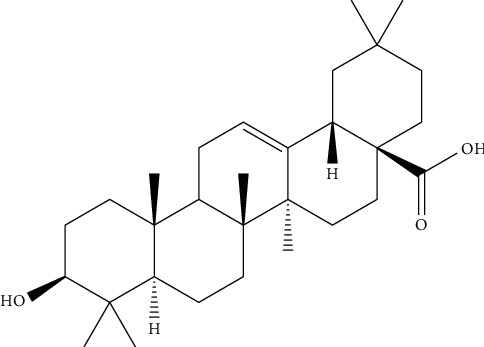
Structure of oleanolic acid.

The antidiabetic activity of *O. tenuiflorum* may be attributed to the presence of metabolites such as oleanolic acid, ursolic acid, and rosmarinic acid, both of which possess strong antidiabetic properties [174, 175]. For reference, OA shown in Figure 8 helps to improve the body's response to insulin and supports the health of pancreatic *β*-cells, which are crucial for insulin production. It also inhibits enzymes such as *α*-amylase and *α*-glucosidase that play a key role in maintaining balanced blood sugar levels. Additionally, OA activates antioxidant pathways, reducing oxidative stress, and blocks inflammatory pathways, both of which are important in managing diabetes and preventing complications [176, 177].

### 4.4. Antifertility Activity

*Ocimum tenuiflorum* is well known for antifertility activity. Mankapure, Mankapure, and Sohani conducted a study on albino rats with doze 400 mg of Tulsi leaves per 100 g of body weight daily for 72 days, which showed a reversible reduction in the testis weight and significant derangements in the histoarchitecture of the testis and epididymis in tested rats [178]. Sethi et al. observed that a doze of 2 g of *O. tenuiflorum* leaves for 30 days resulted in a notable decline in the sperm count, a reduction in follicle-stimulating hormone, and a rise in serum testosterone levels [179]. Similarly, Ahmed et al. concluded that the administration of 250 mg/kg body weight of the benzene extract of *O. tenuiflorum* for 48 days resulted in a lessening of total sperm count, sperm motility, forward velocity, and decreased content of fructose in the caudal plasma of epididymis [180]. This antifertility activity of *O. tenuiflorum* can be attributed to the presence of phytochemicals such as OA and ursolic acid, which are known for antifertility properties [181, 182]. Srinivasulu and Changamma also suggested that ursolic acid acts as an antifertility agent and the study summarized that when the *O. tenuiflorum* leaf extract was administered to rats, it led to a significant decrease in the sperm count and spermatozoa motility by modulating testosterone levels [183].

### 4.5. Anti-Inflammatory Activity

Inflammation occurs when infectious microorganisms invade, reside in tissues, or circulate in the blood and may be triggered by processes such as tissue injury, cell death, cancer, ischemia, and degeneration [184]. The *O. tenuiflorum* as an anti-inflammatory agent has been practiced for a long time. Mirje, Zaman, and Ramabhimaiah found that *O. tenuiflorum* has a superior anti-inflammatory activity compared to the standard anti-inflammatory drug indomethacin in a carrageenan-induced rat paw edema, with administration improving its anti-inflammatory profile [185]. This property may be due to the dual inhibitory property of *O. tenuiflorum* against cyclooxygenase and lipoxygenase [185]. Kaur had synthesized iron nanoparticles using *O. tenuiflorum* to study the anti-inflammatory activity and found that at 100 mg/mL concentration of iron nanoparticles synthesized at 25°C and 0.1 M molarity, the anti-inflammatory activity was maximum of 118.25 [186]. Godhwani, Godhwani, and Vyas observed that the methanol extract and aqueous suspension of *O. tenuiflorum* effectively inhibited inflammation in rats, comparable to the response observed with sodium salicylate with the concentration of 500 mg/kg for prior and 300 mg/kg for later, respectively [187]. Kewlani et al. compared the anti-inflammatory activity of *O. tenuiflorum* and *Azadirachta indica*. In this study, albino rats were injected with formalin to induce inflammation. The samples were administered orally with distilled water, resulting in a remarkable reduction in edema compared to the control group in rats [188]. Similarly, Sharma et al. compared the anti-inflammatory activity of different Ocimum species: *O. basilicum* L., *O. gratissimum* L., and *O. tenuiflorum* L. To evaluate the anti-inflammatory activity, a protein denaturation assay, which is used to induce tissue inflammation, was performed. The results showed that the acetone, methanol, and ethanol extracts of the three Ocimum species significantly protected bovine serum albumin against protein denaturation. The ethanolic extract exhibited the most anti-inflammatory activity, while the water extract of jungle Tulsi and green Tulsi showed the least protection of bovine serum albumin against denaturation [189]. Besides these, essential oil obtained from *O. tenuiflorum* also exhibited strong anti-inflammatory activity by inhibiting the MMP-9 expression in lipopolysaccharide-induced inflammatory cells as per Manaharan et al. [190].

**Figure 9:**
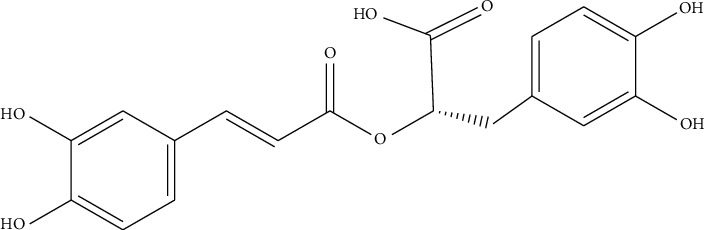
Structure of rosmarinic acid.

The bioactive compounds isolated from *O.tenuiflorum*, particularly rosmarinic acid, eugenol, ursolic acid, apigenin, and luteolin are well documented for their anti-inflammatory activity. Rosmarinic acid (Figure 9) has become well known for its potent anti-inflammatory properties, as supported by numerous studies. It works by suppressing the production of pro-inflammatory cytokines such as tumor necrosis factor-alpha (TNF-*α*) and interleukins (IL-1*β*, IL-6). By inhibiting these cytokines, rosmarinic acid helps reduce inflammation in various inflammatory disease models, such as arthritis and colitis [191].

### 4.6. Antistress Activity

Stress can manifest differently in individuals, and it represents a physiological response that prepares an organism for any action [192]. For relief and freedom from stress, *O. tenuiflorum* is the best medicinal plant. Multiple studies have been conducted to elucidate the antistress properties of *O. tenuiflorum*, and the results consistently indicate a robust antistress effect. Richard et al. explored the antistress property of *O. tenuiflorum* in the chronic variable stress (CVS) model. It demonstrated a concentration-dependent decrease in the cortisol level, i.e., 89% inhibition at 100 *μ*g/mL and 50% inhibition at 6.25 *μ*g/mL. Additionally, the *O. sanctum-*administrated rat's weight increased remarkably compared to the CVS group. This effect on the body weight was attributed to the antistress activity of *O. tenuiflorum* [193]. Similarly, Mohan et al. conducted an in vivo swim endurance study on mice, with an extract of *O. tenuiflorum* escalated the swimming time in the tested sample and lessened the stress-induced increase in immobility time. This result suggested the antistress property of the tested sample [194]. On top of that, Saxena et al. reported the effect of *O. tenuiflorum* to manage stress without causing any side effect [195]. Gupta et al. explored the bioactive compound of *O. tenuiflorum* or the antistress activity and investigated that Ocimumoside A, Ocimumoside B, and 4-allyl-1-O-*β*-D-glucopyranosyl-2-hydroxybenzene are responsible for the antistress property [196].

### 4.7. Anticancer Activity

Due to the presence of secondary metabolites such as flavonoids, sterols, esters, and acyl lipids, plants can be considered a source of anticancer agents [197]. Multiple investigations were carried out to explore the anticancer activity of *O. tenuiflorum*. Boonyanugomol et al. conducted a study on the anticancer activity of *O.tenuiflorum* essential oil against a gastric cancer cell line. They used MTT assays and cell migration and invasion assays to assess cell viability and inhibit metastasis. The results indicated that the viability of AGS cells decreased with an IC_50_ of 163.42 *μ*g/mL when treated with *O.tenuiflorum* essential oil. Furthermore, this treatment induced cellular changes, including cell shrinkage, chromatin condensation, and fragmentation, which are commonly recognized as structural characteristics of apoptotic cell death [198]. Karthikeyan et al. also reported that the ethanolic extract of *O. tenuiflorum* treatment caused a significant reduction in the tumor volume in inoculated sarcoma—180 cells. Along with this, the lifespan of tested animals also increased by 73% for the aqueous extract and 118% for ethanolic extract treatment. These results indicate that for the reduction of tumor development, the ethanolic extract seems to be more effective compared to the aqueous extract [199]. Indrayudha and Hapsari also compared the cytotoxic activity of two plant species: *Cinnamomum burmannii* and *O. tenuiflorum* Linn against T47D cancer cells. For the cell viability, an MTT assay was conducted. From the cytotoxicity tests, it was concluded that *O. tenuiflorum* is more effective compared to *Ci. burmannii* with IC_50_ values of 266.43 and 456.01 *μ*g/mL, respectively [200]. Similarly, Lam, Neda, and Mohd Salleh also conducted the anticancer activity of *O. tenuiflorum* leaves, against human breast cancer cell lines and human fibroblast cell lines, and suggested a remarkable decrease in viability in MCF-7 cells when treated with variable concentrations of the methanolic extract with an IC_50_ of less than 100 *μ*g/mL [201].

**Figure 10:**
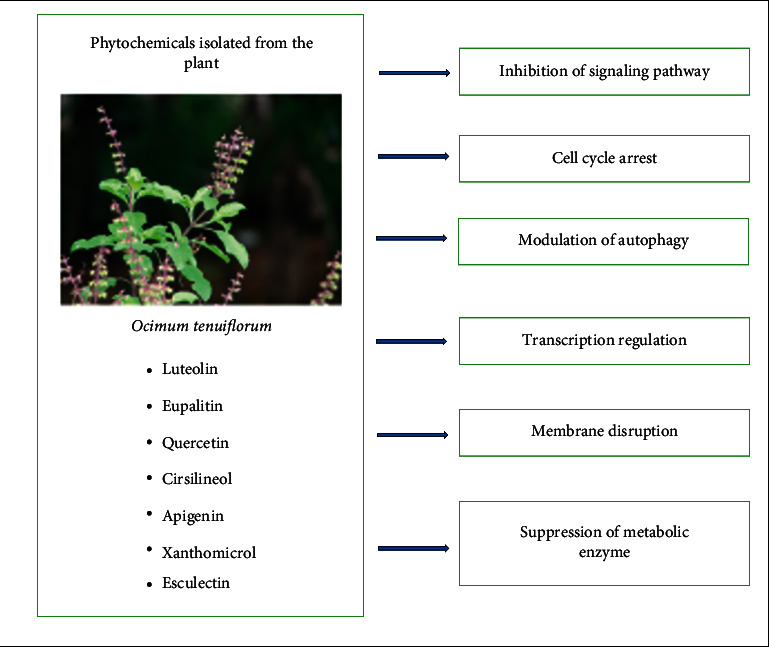
Mechanism of anticancer action of compounds isolated from *Ocimum tenuiflorum*.

The anticancer mechanism of compounds isolated from this plant may be due to different actions such as the inhibition of the signaling pathway, cell cycle arrest, modulation of autophagy, transcription regulation, membrane disruption, and suppression of metabolic enzyme, which is depicted in Figure 10 [202–204].

**Figure 11:**
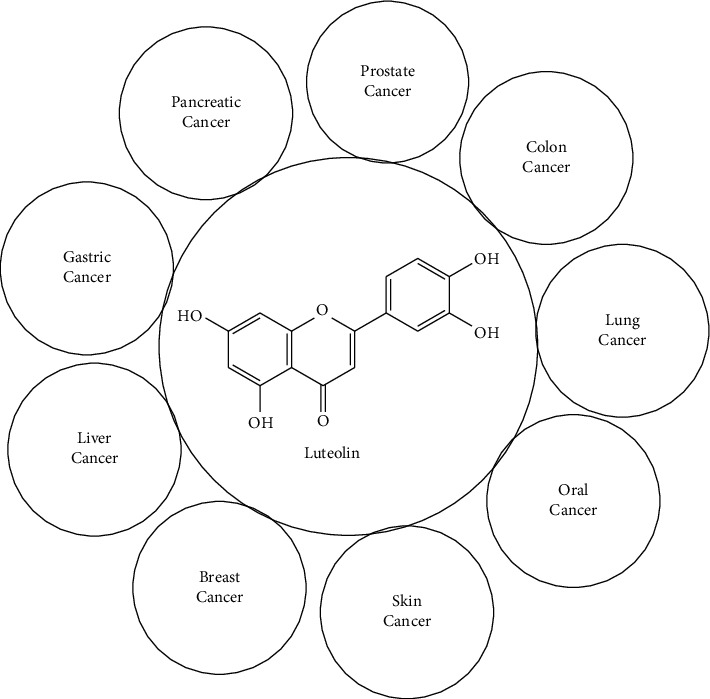
Anticancer activity of luteolin.

For reference, luteolin is a potent bioactive compound that works synergistically with anticancer drugs to inhibit cancer progression. It is effectively used in treating different cancers as shown in Figure 11, including colon, breast, prostate, and liver cancers by inducing apoptosis, arresting the cell cycle, and inhibiting metastasis and angiogenesis. The strength of its anticancer effects comes from its oxidative properties and its ability to interact with multiple targets and signaling pathways in tumor cells, enhancing its overall efficacy [205, 206].

### 4.8. Other Biological Activities

*Ocimum tenuiflorum* has been studied for its multiple biological purposes. It has been reported as a significant wound-healing agent. This test was conducted using incision, excision, and dead space wounds in rats [207]. Extracts and oil obtained from *O. tenuiflorum* were reported to have remarkable analgesic and antipyretic properties [187, 208, 209]. It is also effectively combated against heavy metals, anti-TB drugs, gastric ulcerations, reducing hepatocarcinogenesis, and improving hepatic metabolism [9]. Additionally, the extract containing metabolites such as OA, ursolic acid, rosmarinic acid, eugenol, carvacrol, linalool, and *β*-caryophyllene of *O. tenuiflorum* inhibit COX-2, which is responsible for the inflammation and pain [210]. On the other hand, rosmarinic acid present in *O. tenuiflorum* is reported to be responsible for antiaging activities of the *O. tenuiflorum* [211]. In bovine subclinical mastitis, the aqueous extract of the *O. tenuiflorum* demonstrated immunotherapeutic potential through intramammary infusion, enhancing the phagocytic activity and phagocytic index, reducing total bacterial count, and increasing neutrophil and lymphocyte counts [212]. This plant has been tested for anticonvulsant efficacy, and its ethanol and chloroform extracts from the stem, leaf, and stem callus are potent in suppressing trans corneal electroshock-induced tonic convulsions, comparable to the standard drug phenytoin [213]. Eugenol, an important constituent of *O. tenuiflorum* acts as a strong anthelmintic agent with an ED_50_ of 62.1 *μ*g/mL, which makes plant essential oil effective in the anthelmintic activity [214]. The plant was investigated to explore antithyroid properties and the effects of the *O. tenuiflorum* leaf extract on serum triiodothyronine, and thyroxine showed significant decreases in serum T4 concentrations and no changes in T3 and the ratio between T3 and T4. This suggested that *O. tenuiflorum* exhibited antithyroid properties [215]. *Ocimum tenuiflorum* has been shown to protect against toxicants such as industrial chemicals, pesticides, and pharmaceuticals, preventing liver, kidney, and brain injury. It also protects against the harmful effects caused by acetaminophen, meloxicam, chlorpyrifos, butyl p-hydroxybenzoic acid, copper sulfate, and antitubercular drugs [216–222]. Besides these, *O. tenuiflorum* has been reported to possess properties such as aldose reductase inhibitor, antispasmodic, adaptogenic, cardioprotective, diaphoretic, immune-modulating, anti-inflammatory, antibacterial, antiviral, antifungal, antipyretic, antidiuretic, antidiabetic, antimalarial, and hypolipidemic properties [223–226]. All these properties of *O. tenuiflorum* are due to metabolites present in the plant. Therefore, it is supposed to be an elixir of life.

## 5. Conclusion

*Ocimum tenuiflorum* has long been a cornerstone of traditional medicine, valued for its wide range of therapeutic uses. Even today, its antibacterial, antiviral, antifungal, antipyretic, antidiuretic, antidiabetic, and antimalarial properties are well recognized, largely due to its rich content of metabolites such as flavonoids, phenolics, and terpenoids. Given its potential as a source of antidiabetic, anticancer, antimicrobial, and antioxidant agents, further research into these bioactive compounds is crucial. This includes exploring their design and development, bioavailability, toxicity, and effectiveness, both in their natural state and as derivatives. *Ocimum tenuiflorum* holds promise as a valuable source of bioactive metabolites, offering the potential for the development of novel therapeutic agents in the future.

## Figures and Tables

**Table 1:** Traditional uses of *Ocimum tenuiflorum* in different countries.

Country	Local name	Used parts	Uses	References
Nepal	Tulasi	Leaves	Antioxidant	[15]
India	Tulsi	Leaves	Cough, stomachic, anthelmintic, alleviate muscular pain, joint pain, severe headache	[20, 21]
Saudi Arabia	Shajrat-az-zir	Leaves	Treat coughs, bronchitis	[22]
Bangladesh	Khalatulsi	Leaves	Insect sting, coughing, asthma, fiver	[23, 24]
Thailand	Kaphraodaeg	Leaf	Relieves nausea, stomachache, and flatulence, treats skin disease	[25–27]
Myanmar	Kala-pi-sein, pin-sein-net	Leaf, seed, root	Expectorant and stomachic, kidney diseases, diaphoretic.	[28]
Pakistan	Jungle booti	Leaves/whole plant	Appetizer, mosquito repellent, fodder, fever, cough, headache, diarrhea	[29]

**Table 2:** Biological activity of flavonoids present in *Ocimum tenuiflorum*.

Secondary metabolites	Biological activities	Toxicology and side effect	References
Luteolin	• Antioxidant activity • Antibacterial activity • Anti-inflammatory activity • Anticancer activity • Antidiabetic activity • Antiasthmatic activity • Protect cardiomyocyte cells from LPS-induced apoptosis	• DNA damage • Chromosome damage	[37–40]

Eupalitin	• Antiproliferative against human colorectal tumor cells • Inhibition of the PC3 cell	• Unknown	[41, 42]

Vitexin	• Anticonvulsant effects • Antidepressant effects • Antihypoxia/ischemia injury activity	• Nausea • Headaches • Stomach upset • Skin reactions	[43–46]

Quercetin	• Antioxidant activity • Anticancer activity • Anti-inflammatory activity	• Kidney damage at high doses	[47–50]

Cirsilineol	• Anticancer properties • Antiplatelet agent	• Unknown	[51, 52]

Apigenin	• Anticancer activity • Antioxidant activity • Reduce pulmonary hypertension • Enhance lipid metabolism	• Diarrhea • Skin rashes • Itching • Swelling **•** Difficulty breathing	[53–56]

Xanthomicrol	• Anticancer activities • Antifungal activities • Antioxidant activity	• Unknown	[57, 58]

Orientin	• Antiviral activity against para 3 • Antibacterial activity • Vasodilatation effects • Antinociceptive effects	• Unknown	[59–61]

Chrysoeriol	• Inhibit the induction of nitric oxide synthase by suppressing AP-1 activation • Antioxidant activity • Antimicrobial activity	• Unknown	[62–64]

Esculin	• Anti-inflammatory activity • Antidiabetic activity • Antithrombotic activity • Antibacterial activity	• Gastrointestinal effects • Neurologic effects • Risk of bleeding • Stomach upset • Muscle twitching • Weakness • Vomiting	[65–69]

Esculetin	• Antitumor pharmacological activities against colorectal cancer, gastric cancer, prostate cancer, and breast cancer • Immunomodulatory activity • Antiatherosclerotic activity	—	[65, 70–74]

**Table 3:** Biological activity of phenol and phenolic acids present in *Ocimum tenuiflorum*.

Secondary metabolites	Biological activities	Toxicology and side effect	References
Rosmarinic acid	• Antioxidant and DNA damage protection ability • Restore cognitive functions, anticancer	• No significant toxic effects observed	[79–81]

Chlorogenic acid	• Anti-hepatitis B virus, regulation of carbohydrate and lipid metabolism • Protect liver and kidney • Protect the nervous system	• Overdoses may cause anxiety, agitation, and irregular heartbeat	[82, 83]

Caffeic acid	• Antimicrobial activity • Antioxidant activity	• Overdoses may cause fetal weight gain • Mild stomach upset at higher doses	[84–86]

Vanillin	• Anticancer activity • Antioxidant activity • Protective effects against Huntington's disease • Antisickling agent • Antimicrobial activity	• Mild headaches and allergic reactions	[87–91]

Sinapic acid	• Antiproliferative on colon cancer cells • Antioxidant activity • Antimicrobial activity	• Unknown	[92–94]

*p*-Coumaric acid	• Antinecrotic and anticholestatic effects against liver injury • Antiamoebic activity • Hypopigmenting agent	• Goitrogenic activity	[95–97]

Protocatechuic acid	• Antioxidant • Antibacterial • Antiviral (Control bird flu infection) • Anticancer • Antiosteoporotic • Analgesia • Antiwrinkle properties	• Depletion of GSH in the liver and kidney • LD_50_ 800 mg/kg	[98–104]

Ferulic acid	• Antioxidant • Hepatoprotective • Anticarcinogenic • Antimicrobial, • Antiaging properties • Angiogenic agent	• Unknown	[105–107]

**Table 4:** Biological activities, toxicology, and side effects of terpenoids present in *Ocimum tenuiflorum*.

Secondary metabolites	Biological activities	Toxicology and side effect	References
*β*-Sitosterol	• Anxiolytic effects and sedative effects • Antibacterial activity • Anti-inflammatory • Antioxidant • Antidiabetic • Wound-healing effect	• Mild effects observed such as nausea, indigestion, gas, diarrhea, or constipation • Pancreatitis	[118–120]

Ursolic acid	• Anti-inflammatory property • Anticancer activity • Antibacterial • Antidiabetic • Neuroprotective activity • Herbicidal activity	• Hepatotoxicity • Diarrhea • Nausea • Abdominal swelling • Trace amounts of blood in the urine	[121, 122]

*β*-Sitosterol-3-O*β*-D glucopyranoside	• Potential as a leukemia treatment	—	[123]

Oleanolic acid	• Anticancer activity • Antimicrobial activity • Hepatoprotective effect • Antioxidant activities • Anti-hypertensive activity	• Cholestatic liver injury • Fatigue • Nausea • Anorexia	[124, 125]

**Table 5:** Biological activities, toxicology, and side effects of Monoterpenes present in *Ocimum tenuiflorum*.

Secondary metabolites	Biological activities	Toxicology and side effect	References
*α*-Pinene	• Antibacterial activity • Antifungal activity • Antileishmania activity • Anti-inflammatory activity • Neuroprotective activity • Antiapoptotic activity • Antitumor activity, insecticidal activity	• At 200 *μ*g/mL, BEAS-2B cellular viability decreased • Respiratory and skin irritation	[131–133]

Limonene	• Antibacterial activity • Antioxidant effect • Antidiabetic activity • Anti-inflammatory effect • Anticancer effect • Gastroprotective effect • Antistress effect	• Skin and eye irritation	[134–140]

Estragole	• Anti-inflammatory • Antioxidant • Antibacterial activity	• Genotoxic carcinogen • Hepatocellular adenoma	[141, 142]

Eugenol	• Antioxidant • Antibacterial activity • Anti-inflammatory activity	• LD_50_ value > 1930 mg·kg^−1^ in rodents • Excess use may cause vomiting, gastroenteritis, and systemic toxicity • May cause liver and kidney damage • Seizures • Coma • Bronchial irritation • Dizziness • Rapid breathing	[143–147]

Terpineol	• Antioxidant activity • Anticancer activity • Anticonvulsant activity • Insecticidal activity • Antiulcer activity	• Mild skin irritation or dermatologic allergic response • Eye irritation • Respiratory irritation • Skin irritation • Germ cell mutagenicity • Carcinogenicity, reproductive toxicity	[148, 149]

**Table 6:** Biological activities, toxicology, and side effects of metabolites present in *Ocimum tenuiflorum*.

Secondary metabolites	Biological activities	Toxicology and side effect	References
Phytol	• Antiradical activity • Antibacterial activity • Antifungal activity • Antinociceptive activity	• Decreased mitotic index, and increased DNA damage in the allium cepa test system at certain concentrations • Premature birth • Neonatal cardiovascular abnormalities • Reduced bone mineral density • Damage to lung tissue	[153–158]

Benzeneacetic acid	• Antimicrobial activity	Unknown	[159]
